# The Associations of Pornography Use and Body Image Among Heterosexual and Sexual Minority Men

**DOI:** 10.1007/s10508-024-02887-5

**Published:** 2024-08-07

**Authors:** Ateret Gewirtz-Meydan, Beáta Bőthe, Zohar Spivak-Lavi

**Affiliations:** 1 School of Social Work, Faculty of Social Welfare and Health Sciences, University of Haifa, 3498838 Haifa, Israel; 2 Département de Psychologie, Université de Montréal, Montréal, PQ Canada; 3 Centre de recherche interdisciplinaire sur les problèmes conjugaux et les agressions sexuelles, Montreal, PQ Canada; 4 Faculty of Social Work, Max Stern Yezreel Valley College, Emek Yezreel, Israel

**Keywords:** Pornography use frequency, Problematic pornography use, Social comparison, Body image, Sexual minority men, Sexual orientation

## Abstract

This study investigated the relationship between pornography use and men’s body image, utilizing the social comparison theory as the theoretical framework. The research focused on a moderated mediation model, examining the role of social body comparison as a mediator between pornography use (frequency and problematic use) and men’s body image. The sample consisted of 726 men aged 18–68, with 223 (30.7%) identifying as sexual minorities. Sexual minority men reported higher levels of pornography use frequency, problematic use, perceived realism, social body comparison, negative body image, and psychological distress compared to heterosexual men. Findings revealed that problematic pornography use (but not frequency of use) was related to higher levels of social body comparison, which, in turn, were related to higher levels of negative body image. The perceived realism in pornography did not moderate the examined associations. Clinicians should consider the impact of the relationship between pornography and body image among men.

## Introduction

The current study is based on the established link between social media consumption and body image concerns and dissatisfaction, which holds for both women and men (Holland & Tiggemann, 2016). It has been suggested that the process explaining this relationship is an internalization of body ideals and appearance comparison (Fardouly & Vartanian, 2016; Holland & Tiggemann, 2016; Ryding & Kuss, 2020). While the association between social media use and body image concerns and dissatisfaction has received much empirical attention, the association between pornography use and body image has received far less attention. Like social media, pornography is highly available and prevalent, especially among men (Grubbs et al., 2019; Herbenick et al., 2020; Rissel et al., 2017). While historically, pornography was consumed by images in magazines or videotapes, nowadays pornography is consumed mostly through the internet. Considering the “triple A” influence (accessibility, affordability, anonymity), online pornography is on the rise (de Alarcón et al., 2019). Studies suggest that pornography, characterized by an extreme portrayal of sexual intimacy and unrealistic beauty standards, often objectifies individuals, reinforcing societal beauty norms that are challenging for the average person to attain, contributing to growing insecurities and comparisons, ultimately leading to low body image (Dawson et al., 2020; Paslakis et al., 2022). The current study sought to examine how pornography use and problematic pornography use might relate to men’s body image, using a moderated mediation model.

### Social Comparison as the Theoretical Framework of the Study

Social comparison is a form of sociological self-esteem, where the sense of self is developed through comparing oneself with others (Festinger, 1954). Festinger argued that people have an innate drive to evaluate themselves, and they determine their own social and personal worth based on how they measure up against others. There are two main types of social comparison: downward and upward. Downward social comparisons involve comparing oneself to someone else perceived as “lesser” or “worse” (i.e., with those who are worse off or less skilled than them) and upward social comparisons, which involve comparing oneself to someone else that is perceived as “better” (Tiggemann & Polivy, 2010). In a social media context, people often engage in upward social comparisons (e.g., viewing others on social media as more attractive than them), a process that is associated with reduce self-esteem, and greater body image concerns (Hargreaves & Tiggemann, 2009; Tiggemann & Anderberg, 2020b).

Lacking an objective basis for determining one’s own level of physical attractiveness, people often evaluate themselves relative to available social standards, including the standards of appearance presented in the media. Using the social comparison theory, studies have explained the association between media consumption and low body image (Myers & Crowther, 2009; van den Berg et al., 2007). Namely, ideal images are produced in the media after arranging the lighting, getting the best angle, taking multiple photographs, and applying filters or Photoshop, resulting in unreal images (Tiggemann & Anderberg, 2020b). When individuals compare themselves to idealized and, in many cases artificially created images, they are likely to feel frustrated and dissatisfied with their own appearance (Want, 2009). The process of upward comparison with ideal images in the media has also been associated with poorer mental health and psychological well-being (Hanna et al., 2017; Jang et al., 2016; Tiggemann & McGill, 2004), possibly because positively framed information of comparison objects gives rise to upward comparison, which in turn evokes a sense of relative deprivation or distress, aggravating one’s mental health (Jang et al., 2016).

Although the association between consuming social media and low body image has been well-established and theoretically conceptualized, gaps in the literature still remain. Most research on body image has focused on women (Grabe et al., 2008), even though men can be also susceptible to media messages about ideal bodies (Barlett, 2008; Dittmar et al., 2009). The majority of research on the association between social media and body image (Fardouly & Vartanian, 2016; Holland & Tiggemann, 2016; Ryding & Kuss, 2020), with little attention given to understanding the association between pornography use and body image, despite pornography being consumed by the vast majority of men (Miller et al., 2020). While pornography is highly available and prevalent just like social media, it is an extreme portrayal of sexual intimacy and unrealistic beauty standards, which often objectifies individuals by displaying their intimate body parts under the spotlight. As such, in the current study, we examined the association of pornography use frequency and problematic pornography use, with body image, considering the mediating role of social body comparison among men.

According to a recent review, most (> 80%) adult men have accessed pornography at some point in their life, and over the past year (40–70%). Further, around half of younger men (25 or under) are weekly consumers (Miller et al., 2020). Recent studies looking at trends in pornography use indicate that pornography use (on the Internet) is increasing consistently over time (Lewczuk et al., 2022a, 2022b). Recent data from the International Sex Survey (Beaulie, 2023) show that 97.1% of men, 85% of women and 93.7% of gender-diverse individuals have reported lifetime pornography use. However, using pornography does not necessarily indicate problematic use (Beaulieau, 2023; Bőthe et al., 2020).

As for problematic pornography use, approximately 6% of pornography users report problematic pornography use (i.e., uncontrollable patterns of pornography use resulting in significant distress and adverse consequences), with it being more prevalent among men (Grubbs et al., 2020). In a recent study, Bőthe et al., 2023 conducted among 82,243 people from 42 countries reported problematic pornography use. Specifically, 3.2% of participants scored above the ProblematicPornography Consumption Scale (PPCS) cut-off, 9.8% exceeded the PPCS-6 cut-off, and 16.6% surpassed the Brief Pornography Screen (BPS) cut-off.

Although lacking a consensual definition (Short et al., 2012), pornography can be defined as materials deemed sexual that have the primary intention of sexually arousing the consumer (Carroll et al., 2008; Hald, 2006; Kohut et al., 2020; Lam & Chan, 2007; McKee et al., 2020). Pornography contains images of bodies that are often idealized, featuring actors whose body dimensions and proportions are far from those of the general population in terms of muscularity, body fat, height, and the size and shape of genitalia and other body features (Dawson et al., 2020). While pornography showcases a diversity of female bodies, including petite and large bodies, small and large breasts, male performers tend to adhere to a more uniform standard characterized by muscularity and well-endowed attributes. Consequently, the physical appearance of male performers exhibits less diversity compared to their female counterparts in pornography (McKee et al., 2008). While previous research examined the association between pornography use and male’s body image (Paslakis et al., 2022), the potential mechanism (e.g., social body comparison) explaining the associations of pornography with men’s body image has not received as much attention.

In a recent systematic review (Paslakis et al., 2022) examining the association between pornography and body image through qualitative and quantitative studies, researchers found consistent evidence of negative impacts. Qualitative studies revealed that women often held critical views, suggesting that pornography reinforces unrealistic societal beauty standards, including slim bodies and specific genital representations (Dawson et al., 2020; Leickly et al., 2017). Men, acknowledging the larger-than-average penis size of pornography actors (Sharp & Oates, 2019), exhibited associations between frequent pornography use and penis size dissatisfaction (Cranney, 2015; Sharp & Oates, 2019). Quantitative studies indicated that increased exposure to pornography correlated with negative body attitudes, greater body dissatisfaction, lower physical self-esteem, heightened body surveillance, and increased internalization of appearance ideals (e.g., Goldsmith et al., 2017; Griffiths et al., 2018; Peter & Valkenburg, 2014; Sevic et al., 2020; Tylka, 2015; Whitfield et al., 2018). Other associations included a higher drive for muscularity, more frequent thoughts about using anabolic steroids, and increased eating disorder symptomatology (e.g., Griffiths et al., 2018). However, according to this review, certain studies revealed no significant associations, or even a positive one (Duggan & McCreary, 2004; Kvalem et al., 2014; Vogels, 2019). One of the possible explanations for these mixed findings can be based on how excessively pornography is used. The current study draws on previous findings that show problematic pornography use has a greater negative effect on a person’s life, whereas non-problematic viewing of pornography appears to have little or no negative impact on a person’s life and overall functioning (Bőthe et al., 2018, 2020; Chen et al., 2022; Kraus et al., 2018). The excessiveness, the uncontrollable viewing, and for some—acceleration in use can all explain why problematic pornography use is more impactful on a person’s life. Given this distinction, our study places a strong emphasis on problematic pornography use. It is acknowledged that body dissatisfaction is not solely influenced by the quantity of exposure to pornography, but also by psychological elements in problematic use, such as craving and withdrawal (Lewczuk et al., 2022a, 2022b). While frequency captures exposure, we argue that problematic use provides a more comprehensive understanding of the complex link between pornography use and body dissatisfaction. This is because the key cause of body dissatisfaction goes beyond the degree of exposure to unrealistic bodies; problematic use includes psychological processes intensifying the impact of exposure and offers a more comprehensive insight into the relationship between pornography use and body dissatisfaction.

Moreover, a growing body of literature suggests that the potential effect of pornography is most significant when viewers have higher perceived realism, meaning when viewers perceive what they see as real and authentic. Perceived realism is often overlooked in pornography research, but the question of viewers’ perceptions of whether what they are viewing as “real” or not is central to the discussion about what people are supposed to learn from pornography and how they are ostensibly “affected” by viewing pornography (Taylor, 2022). In other words, it is assumed that when pornographic images are seen as valid and authentic, it may increase the process of engaging in social comparison, which in turn, might result in worse body image (Tiggemann & Anderberg, 2020b). Previous research has used realism perception to explain the association between pornography use and sexual attitudes toward sex (Baams et al., 2015; Koletić, 2017; Peter & Valkenburg, 2006), sexual behavior (Wright et al., 2022), and sexual aggression (Krahé et al., 2022). A previous study conducted among 393 men and women examined the link between pornography use and body image, and unexpectedly found a positive indirect association between pornography use and body image through perceived realism for both men and women (Vogels, 2019). The current study adds to the examination of perceived realism to obtain a more nuanced examination of the links between pornography and body image, and pornography and social comparison, as social comparison and body image disturbances are developed even and perhaps, especially when the images are unrealistic (highly enhanced or edited photos) but still perceived realistic by the viewer (Tiggemann & Anderberg, 2020b). The current study sought to shed light on the association between pornography use and body image and the mechanism explaining this association, by examining the mediating role of social body comparison and the moderating role of perceived realism in the associations between pornography use frequency, problematic pornography use, and body image.

### Differences Between Sexual Minority and Heterosexual Men

Studies have indicated that men from sexual minorities report higher levels of pornography use (Downing et al., 2017; Rissel et al., 2017), body image disturbances (Tiggemann et al., 2007), and poorer mental health. According to the intra-minority stress theory (Pachankis et al., 2020), the elements of status within the sexual minority community are based on masculinity, attractiveness, and wealth. In addition to intra-community pressures, individuals who identify as sexual minority also experience more external stressors such as discrimination and violence, as outlined by the minority stress model (Meyer, 2003). Thus, it is not surprising that sexual minority men are more likely than heterosexual men to report dissatisfaction with their physical appearance and muscle size/tone, and to agree that they experienced objectification, surveillance, appearance-based social comparison, and pressure from the media to be attractive (Frederick & Essayli, 2016). Based on indications from the literature and grounded in both the intra-minority stress theory (Pachankis et al., 2020), and the sexual minority stress model (Meyer, 2003), the current study oversampled men from sexual minorities to test differences between heterosexual and sexual minority men in the association of pornography use, social comparison, and body image, and examine the moderating role of perceived realism in these associations.

### The Present Study

Although an extensive amount of research has examined body image and social comparison in relation to social media (Saiphoo & Vahedi, 2019; Tiggemann & Anderberg, 2020a), most studies have focused on women (Grabe et al., 2008). Far less attention has been given to understanding the association of pornography use with body image, despite pornography being consumed by the vast majority of men (Miller et al., 2020). Like social media, pornography is highly available and prevalent, particularly among men. However, it is an extreme portrayal of sexual intimacy and unrealistic beauty standards, which often objectifies individuals by displaying their intimate body parts under the spotlight. This high objectification of the body and intimate sexual parts may contribute to growing insecurities and comparisons among consumers. In the current study, we aimed to examine the associations of pornography use frequency, problematic pornography use, social body comparison, and men’s body image*,* considering the moderating role of perceived realism. We hypothesized that problematic pornography use, but not frequency of pornography use, would be associated with greater social body comparison, which in turn, would be associated with more negative body image. We also hypothesized that these associations would be stronger among those individuals who have higher levels of perceived realism in pornography. Based on previous research indicating that men from sexual minorities report higher levels of pornography use (Downing et al., 2017; Rissel et al., 2017) and body image disturbances (Tiggemann et al., 2007), we examined the model among both heterosexual and sexual minority men to explore potential differences between these groups. Given that depression and anxiety are prevalent among individuals with problematic pornography use (Kraus et al., 2015), and are associated with lower body image among men (Barnes et al., 2020), psychological distress (operationalized as depressive and anxiety symptoms) was included as a control variable in our model. Nevertheless, considering recent calls highlighting the shortcomings of using control variables in pornography use studies (Wright, 2021), we reported our findings both with and without the inclusion of the control variable for full transparency.

## Method

### Participants

A total of 726 men participated in the study. The men ranged in age from 18–68 with an average age of 32.50. Most of the men defined themselves as heterosexual, but sexual minority men were oversampled in the study and comprised approximately one-third of the sample. Men who identified as sexual minority defined themselves as gay (13.2%), heteroflexible (7.1%), homoflexible (2.7%), bisexual (5.7%), queer (0.1%), pansexual (0.6%), or other (1.1%). The majority of the sample were Jewish, non-religious, educated, working full time, in a relationship, with no children. Full sample characteristics are in Table 1.

**Table 1:** Sociodemographic characteristics of the sample (n = 726)

Measure	N (%)
Age (in years) (M, SD)	32.5 (9.22)
Heterosexual	491 (67.6)
Sexual minority	233 (32.4)
Transgender	3 (0.7)
Education	
Primary (e.g., elementary school)	6 (0.8)
Secondary (e.g., high school)	231 (31.8)
High (e.g., college or university)	489 (67.4)
Work	
No	97 (13.4)
Yes, full time	502 (69.1)
Yes, I do sporadic jobs	29 (4)
Yes, part-time	98 (13.5)
Relationship status	
In relationship	448 (61.7)
Not in relationship	279 (38.3)
Have children	
No	470 (64.7)
Religion	
Jewish	658 (90.6)
Muslim	3 (0.4)
Christian	5 (0.7)
Other	60 (8.3)
Religiosity	
Non- religious	513 (70.7)
Traditional^a^	101 (13.9)
Religious	65 (9)
Very religious (ultra-orthodox)	15 (2.1)
Other	32 (4.4)

^a^In Israel, the differentiation between “traditional” and “religious” subcategories of religiosity is often based on the level of adherence to Jewish laws, with “traditional” individuals identifying culturally but not strictly observing, reflecting the diverse spectrum of Jewish identity

### Procedure

The study, conducted in Israel, utilized paid ad campaigns on social media platforms, specifically Facebook and Instagram, to reach the intended audience. Invitations were extended to individuals, primarily men, to participate in a study on body image and eating habits. The oversampling of sexual minority men was achieved through targeted techniques on social media. Inclusion criteria were identifying as a man aged 18 or over who could read Hebrew. The survey took an average of 20 min to complete and was open from September 2021 through January 2022. The study was anonymous, and no data were collected that linked participants to recruitment sources. Clicking on the link to the survey guided potential respondents to a page that provided information about the purpose of the study, the nature of the questions, and a consent form (the survey was voluntary and respondents could quit at any time). The first page also offered the researchers’ contact information. After completing the survey, each participant could enter a lottery to win one of five $85 gift vouchers.

### Measures

Frequency of pornography use was assessed by asking participants how often they had consumed pornography in the last six months. Response options (using a six-month recall period) ranged from 1 = never to 7 = every day or almost every day. These response options are similar to those used in prior research (Bridges et al., 2016; Sun et al., 2017; Wright & Štulhofer, 2019).

Problematic pornography use was measured using the Problematic Pornography Consumption Scale-Short Version (PPCS-6; (Bőthe et al., 2021a, 2021b, 2021c). The short version of the PPCS-6 assesses problematic pornography use with six items. Participants indicated their answers on a 7-point scale, ranging from 1 (never) to 7 (all the time) regarding the past 6 months. Higher scores indicate higher levels of problematic pornography use. In the current study, the scale had good reliability (α = 0.84; ω = 0.84).

Perceived realism was measured using Peter and Valkenburg’s (Peter & Valkenburg, 2006) perceived realism of pornography use scale. This scale consists of four items assessing how realistic individuals perceive pornography to be. Participants rated the extent to which they agreed with statements using a 6-point Likert scale ranging from 1 (I totally disagree) to 5 (strongly agree). Items were summed to create a total score. In the current study, the scale had acceptable reliability (α = 0.76; ω = 0.75).

Body image was measured using the Male Body Attitudes Scale (MBAS; (Tylka et al., 2005). The MBAS is a 24-item self-report instrument that measures men’s attitudes toward their bodies. Items are responded to via a 6-point scale, which ranges from 1 (never) to 6 (always), with higher scores denoting more body dissatisfaction. Reliability of the scale in the current sample was excellent (α = 0.94; ω = 0.93).

Social body comparison was measured using the Upward Appearance Comparison Scale (UPACS; (O’Brien et al., 2009)). The UPACS is a 10-item measure assessing individuals’ general tendency to compare their appearance with the appearance of others whom they perceive to be better looking than they are. Items are rated on a 5-point scale from 1 (strongly disagree) to 5 (strongly agree). Items were averaged, with higher scores reflecting a greater tendency to compare one’s appearance with that of others. In the current study, the UPACS had excellent reliability (α = 0.95; ω = 0.95).

Psychological distress was measured using the Patient Health Questionnaire-4 (PHQ-4; (Kroenke et al., 2009) which includes the measurement of two variables: anxiety and depressive symptoms. The scale ranged from 0 (not at all) to 3 (nearly every day). Items were combined to create a total score with higher scores representing more psychological distress. Reliability of the scale in the current sample was good (α = 0.87; ω = 0.87).

Sexual orientation was assessed based on the scale provided by (Weinrich, 2014). Participants were asked to indicate the expression that best described their current sexual orientation (heterosexual or straight; gay or lesbian or homosexual; heteroflexible; homoflexible; bisexual; queer; pansexual; asexual; I do not know yet or I am currently questioning my sexual orientation; none of the above; I don’t want to answer). We created two groups based on sexual orientation to increase statistical power: individuals who reported being heterosexual (*n* = 491, 67.6%) were included in the heterosexual group, and individuals reporting any other sexual orientations (*n* = 233, 32.1%) were included in the sexual minority group. Two participants (0.3%) did not report their sexual orientation, and therefore were not included in either group.

Background variables included questions about age, religion, religiosity, work status, income, children, relationship status and duration, and level of education.

### Data Analysis

Descriptive statistics, reliability indices (i.e., Cronbach’s alphas and McDonald’s omegas), normality indices (i.e., skewness and kurtosis values), and bivariate correlations were computed in SPSS 28. In line with previous studies on body image and sexuality (e.g., Girouard et al., 2021; Paquette et al., 2022), two sets of path analyses were performed to examine the associations of pornography use frequency, problematic pornography use, and body image, considering the mediating role of social body comparison and the moderating role of perceived realism in pornography, with and without controlling for psychological distress (operationalized as depressive and anxiety symptoms), using M*plus* 8.7. Due to the naturally non-normal distribution of the data, we estimated the models using the robust-maximum-likelihood (MLR). Commonly used goodness-of-fit indices were observed to assess the acceptability of the examined models: Tucker–Lewis index (TLI; ≥ 0.90 acceptable; ≥ 0.95 excellent), Comparative Fit Index (CFI; ≥ 0.90 acceptable; ≥ 0.95 excellent), and root-mean-square error of approximation (RMSEA; ≤ 0.08 adequate; ≤ 0.06 excellent) with its 90% confidence intervals. Following prior guidelines (Newman, 2014), the full information maximum likelihood (FIML) method was used to handle missing data.

In the first set of analyses, we started with examining the associations of pornography use frequency, problematic pornography use, and body image, considering the mediating role of social body comparison without the control variable in the total sample (Model 1a). Next, we examined whether this model varied based on sexual orientation (i.e., heterosexual vs. sexual minority men) using multi-group path analysis (Model 1b). In the final step, we tested whether the examined associations differed significantly by constraining the paths and correlations between the variables to be equal across the two groups (Model 1c). When comparing Model 1b and Model 1c (i.e., unconstrained and constrained models), changes in chi-square, CFI, TLI, and RMSEA values were observed. A significant corrected chi-square difference test, significant decreases in CFI and TLI (ΔCFI ≤ 0.010; ΔTLI ≤ 0.010), and significant increases in RMSEA (ΔRMSEA ≤ 0.015) (Bőthe et al., 2021c; Chen, 2007; Cheung & Rensvold, 2002) indicated whether the constrained and unconstrained models differed significantly (i.e., whether the associations differed significantly between heterosexual and sexual minority men). After choosing the final model, we added perceived realism in pornography as a moderator (Model 1d).

In the second set of analyses, we added psychological distress as a control variable to the models and followed the same sequence of model testing. We examined examine the associations of pornography use frequency, problematic pornography use, and body image, considering the mediating role of social body comparison, with the control variable in the total sample (Model 2a); examined whether this model varied based on sexual orientation status using multi-group path analysis (Model 2b) and tested whether the associations differed significantly between these groups (Model 2c) by observing the changes in the previously described fit indices. After choosing the final model, we added perceived realism in pornography as a moderator (Model 2d).

Following prior recommendations, indirect effects in the mediation models were tested via the calculation of bias-corrected bootstrap (10,000 bootstrap replication samples) 95% confidence intervals (CI; Frederick & Essayli, 2016; Preacher & Hayes, 2008; Schellenberg et al., 2016). Bonferroni correction was applied (α = 0.05; m = 5, as 5 moderations were included in each model) to reduce the risk of type I error in the examined moderations. Consequently, moderations were considered significant at *p* < 0.010.

## Results

### Descriptive Statistics and Comparisons of Heterosexual and Sexual Minority Men

Descriptive statistics, reliability indices, and associations of pornography use frequency, problematic pornography use, and perceived realism in pornography, with social body comparison, body image, and psychological distress, are shown in Tables 2 and 3. Sexual minority men reported significantly higher levels of pornography use frequency, problematic pornography use, perceived realism in pornography, social body comparison, negative body image, and more psychological distress than did heterosexual men, with small to medium effect sizes (Table 4).

**Table 2:** Descriptive statistics and correlations between all study variables

Variables	Skewness (*SE*)	Kurtosis (*SE*)	Range	*M* (*SD*)	1	2	3	4	5
1. Pornography use frequency^a^	−0.36 (0.09)	−1.07 (0.18)	1–7	4.64 (1.91)	–				
2. Problematic pornography use	0.94 (0.09)	0.57 (0.18)	6–42	15.39 (7.54)	0.55**	–			
3. Perceived realism in pornography	0.81 (0.09)	0.44 (0.18)	4–20	7.93 (3.20)	0.19**	0.25**	–		
4. Social body comparison	0.44 (0.09)	−0.44 (0.18)	10–50	24.62 (9.89)	0.14**	0.29**	0.30**	–	
5. Negative body image	0.11 (0.09)	−0.67 (0.18)	29–144	82.50 (25.31)	0.13**	0.26**	0.22**	0.66**	–
6. Psychological distress	1.00 (0.09)	0.41 (0.18)	4–16	7.50 (3.06)	0.16**	0.31**	0.17**	0.32**	0.37**

*Note SE* = standard error; *M* = mean; *SD* = standard deviation

^a^ 1 = never, 2 = a few times, 3 = once a month, 4 = 2–3 times a month, 5 = once a week, 6 = a few times a week, 7 = every day or almost every day

*p < 0.05; ** *p* < 0.001

**Table 3:** Descriptive statistics and correlations between all study variables among heterosexual and sexual minority men

Variables	1	2	3	4	5	6
1. Pornography use frequency	–	0.55**	0.17**	0.09	0.10	0.18**
2. Problematic pornography use	0.55**	–	0.21**	0.22**	0.16*	0.28**
3. Perceived realism in pornography	0.18**	0.25**	–	0.35**	0.26**	0.13*
4. Social body comparison	0.12**	0.29**	0.23**	–	0.70**	0.20**
5. Negative body image	0.12**	0.28**	0.15**	0.61**	–	0.31**
6. Psychological distress	0.14**	0.30**	0.14**	0.34**	0.38**	–

*Note* Correlations above the diagonal represent results among sexual minority men, while correlations below the diagonal represent results among heterosexual men

*p < 0.05; ** *p* < 0.001

**Table 4:** Comparisons of heterosexual and sexual minority men along all study variables

Variables	Heterosexual men *Median / M (SD)*	Sexual minority men *Median / M (SD)*	Mann–Whitney *U*-test / Independent samples *t*-test ^a^	*p*	*d*
1. Pornography use frequency	5	6	*U* = 64,734.00	0.004	0.21
2. Problematic pornography use	14.63 (7.07)	16.94 (8.24)	*t*(399.31) = −3.70	< 0.001	0.31
3. Perceived realism in pornography	7.52 (3.02)	8.76 (3.36)	*t*(722) = −4.98	< 0.001	0.40
4. Social body comparison	22.67 (9.17)	28 (10.13)	*t*(722) = −8.07	< 0.001	0.64
5. Negative body image	78.61 (24.06)	90.56 (26.05)	*t*(722) = −6.08	< 0.001	0.48
6. Psychological distress	7.18 (2.96)	8.14 (3.17)	*t*(722) = −3.98	< 0.001	0.32

*Note M* = mean; *SD* = standard deviation

^a^ We used the Mann–Whitney U test to compare the groups regarding the ordinal variables (i.e., pornography use frequency), while we used independent sample *t* tests to compare the groups regarding the continuous variable (e.g., body image)

### The Mediating Role of Social Body Comparison in the Associations of Pornography Use and Body Image without the Control Variable

**Fig. 1:**
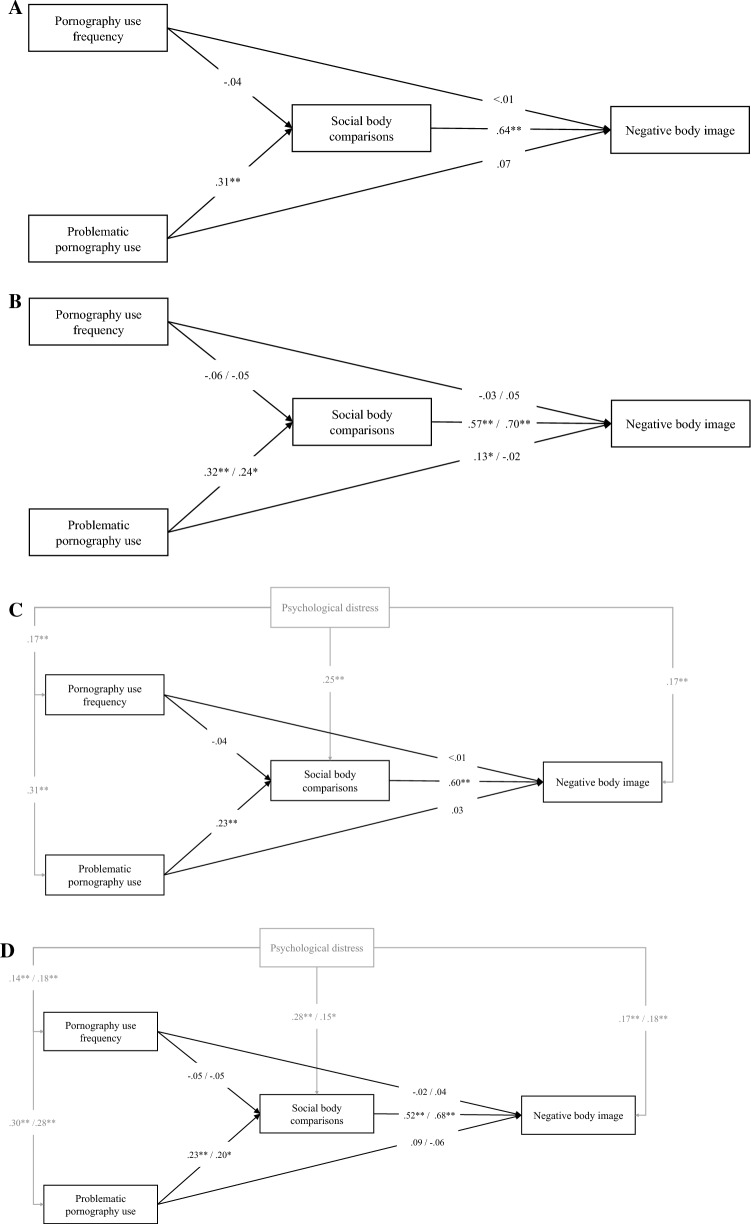
Associations of pornography use frequency, problematic pornography use, social body comparison, and body image, with and without psychological distress as a control variable. **A** Total sample, without the control variable (Model 1A), **B** Heterosexual and sexual minority men, without the control variable (Model 1B), **C** Total sample, with the control variable (model 2A), **D** Heterosexual minority men, with the control variable (Model 2B). *Note* One-headed arrows represent standardized regression weights. Correlations between pornography use frequency and problematic pornography use are not depicted in the figure for the sake of clarity. Numbers on the arrows indicate the standardized path coefficients. The control variable and its associations are depicted with gray. The first numbers on the arrows indicate the path coefficients for heterosexual men, and the second numbers indicate the path coefficients for sexual minority men. Perceived realism in pornography did not moderate any of the examined associations. * *p* < 0.05; ** *p* < 0.01

In the first set of analyses, we examined the associations of pornography use frequency, problematic pornography use, social body comparison, and body image, without the control variable. In the path analysis for the total sample (Model 1a; CFI = 1.00, TLI = 1.00, RMSEA = 0.00 [90% CI 0.00, 0.00]), pornography use frequency was not related significantly to social body comparison, while problematic pornography use had a positive, weak-to-moderate association with social body comparison. Moreover, neither pornography use frequency nor problematic pornography use was directly related to negative body image. However, social body comparison had a positive, strong association with negative body image (see Fig. 1a).

Next, we examined this model across the previously established groups of heterosexual and sexual minority men. To examine whether the identified associations were significantly different between the groups, we compared the unconstrained model (Model 1b; CFI = 1.00, TLI = 1.00, RMSEA = 0.00 [90% CI = 0.00, 0.00]) to a model in which all associations of pornography use frequency, problematic pornography use, social body comparison, and body image were constrained to be equal between the two groups (Model 1c; CFI = 0.991, TLI = 0.985, RMSEA = 0.041 [90%CI 0.000, 0.086]). Although the corrected chi-square difference test (Δχ^2^ = 9.568, *p* = 0.144) did not indicate a significant difference between the models, the changes in the fit indices (ΔCFI = -0.009; ΔTLI = -0.015; ΔRMSEA =  + 0.041) indicated a significant difference between the unconstrained (Model 1b) and the fully constrained (Model 1c) models, suggesting that the associations may differ significantly between heterosexual and sexual minority men.

In the case of heterosexual men, pornography use frequency was not related significantly to social body comparison. However, problematic pornography use had positive, moderate association with social body comparison. Moreover, pornography use frequency was not directly related to negative body image either, while problematic pornography use had a weak, positive association with negative body image. Social body comparison had a positive, strong associations with negative body image. One indirect pathway was significant. Higher levels of problematic pornography use were associated with greater social body comparison, which, in turn, was associated with higher levels negative body image (indirect path’s *β* = 0.18, 95% CI = [0.12, 0.25], p < 0.001). Perceived realism in pornography did not moderate any of the examined associations (*p*s > 0.169).

In the case of sexual minority men, pornography use frequency was not related significantly to social body comparison. However, problematic pornography use had positive, weak-to-moderate association with social body comparison. Neither pornography use frequency, nor problematic pornography use had direct associations with negative body image. Social body comparison had a positive, strong association with negative body image. One indirect pathway was significant. Higher levels of problematic pornography use were associated with greater social body comparison, which, in turn, was associated with higher levels of negative body image (indirect path’s *β* = 0.17, 95% CI = [0.06, 0.28], *p* = 0.002). Perceived realism in pornography did not moderate any of the examined associations (*p*s > 0.054), see Fig. 1b.

### The Mediating Role of Social Body Comparison in the Associations of Pornography Use and Body Image with the Control Variable

In the second set of analyses, we examined the associations of pornography use frequency, problematic pornography use, social body comparison, and body image, with the control variable (i.e., psychological distress) included in the models. In the path analysis for the total sample (Model 2a; CFI = 1.00, TLI = 1.00, RMSEA = 0.00 [90%CI 0.00, 0.00]), pornography use frequency was not related significantly to social body comparison, while problematic pornography use had positive, weak association with social body comparison. Neither pornography use frequency nor problematic pornography use was directly related to negative body image. However, social body comparison had a positive, strong association with negative body image (Fig. 1c).

Next, we examined this model across the groups of heterosexual and sexual minority men. To examine whether the identified associations were significantly different between the groups, we compared the unconstrained model (Model 2b; CFI = 1.00, TLI = 1.00, RMSEA = 0.00 [90% CI = 0.00, 0.00]) to a model in which all associations of pornography use frequency, problematic pornography use, social body comparison, and body image were constrained to be equal between all groups (Model 2c; CFI = 0.995, TLI = 0.982, RMSEA = 0.042 [90%CI 0.000, 0.088]). Although the corrected chi-square difference test (Δχ^2^ = 9.861, *p* = 0.131) did not indicate a significant difference, the changes in the fit indices (ΔCFI = -0.005; ΔTLI = -0.018; ΔRMSEA =  + 0.042) indicated a significant difference between the unconstrained (Model 2b) and the fully constrained (Model 2c) models, suggesting that the associations may differ significantly between heterosexual and sexual minority men.

In the case of heterosexual men, pornography use frequency was not related significantly to social body comparison. However, problematic pornography use had positive, weak-to-moderate association with social body comparison. Neither pornography use frequency, nor problematic pornography use had direct associations with negative body image. Social body comparison had a positive, strong associations with negative body image (Fig. 1d). One indirect pathway was significant. Higher levels of problematic pornography use were associated with greater social body comparison, which, in turn, was associated with higher levels negative body image (indirect path’s *β* = 0.12, 95% CI = [0.06, 0.19], *p* < 0.001). Perceived realism in pornography did not moderate any of the examined associations (*p*s > 0.123).

In the case of sexual minority men, pornography use frequency was not related significantly to social body comparison. However, problematic pornography use had positive, weak-to-moderate association with social body comparison. Neither pornography use frequency, nor problematic pornography use had direct associations with negative body image. Social comparison had a positive, strong associations with negative body image (Fig. 1d). Importantly, the association between social body comparison and negative body image was stronger in the case of sexual minority men than heterosexual men, based on the effect sizes and the not overlapping confidence intervals of the betas. One indirect pathway was significant. Higher levels of problematic pornography use were associated with greater social body comparison, which, in turn, was associated with higher levels negative body image (indirect path’s *β* = 0.14, 95% CI = [0.03, 0.24], *p* = 0.012). Perceived realism in pornography did not moderate any of the examined associations (*p*s > 0.020), considering the pre-established Bonferroni correction for the moderation analyses (*p* < 0.010).

## Discussion

Findings from the current study indicated that problematic pornography use, but not frequency of pornography use, was significantly and directly related to social body comparison and indirectly to negative body image. Our findings correspond with previous studies indicating that problematic pornography use should be distinguished from non-problematic pornography use, given the greater negative impact that problematic use has on users’ lives (Binnie & Reavey, 2020; Bőthe et al., 2018, 2020; Chen et al., 2022; Kraus et al., 2018). Important sexual orientation-based differences were also observed.

Given that pornography typically portrays male models who are muscular and hyper-masculine, it is reasonable to assume that excessive pornography use would be associated with higher levels of negative body image among men. Indeed, this study’s findings support findings from earlier studies indicating that pornography use might be associated with higher levels of negative body image via social comparisons (Paslakis et al., 2022). Moreover, our findings also corroborate results from previous studies indicating no significant associations between pornography use frequency and body image (Duggan & McCreary, 2004; Gleason & Sprankle, 2019; Sevic et al., 2020).

The current study was conducted on the basis of social comparison literature, originally proposed by Festinger (1954), wherein individuals tend to compare themselves to others along various dimensions. Although pornography provides men with many aspects to compare, including body ideals and sexual behaviors, research examining how social comparison explain the associations between pornography use and body image is scarce. The idealized images of the male body presented in pornography may increase comparison-making, and these comparisons usually produce a decrease in self-ratings of the object being compared. For example, two studies found that pornography use seemed to influence men’s perceptions of their penis size. Although men discussed being highly aware that pornography actors had a penis size that was larger than average (Sharp & Oates, 2019), penis size dissatisfaction was found to be associated with pornography use frequency (Cranney, 2015), and men reported that pornography skewed their perceptions of the size range of a normal penis (Sharp & Oates, 2019). Another study found that men who watched more pornography were dissatisfied with their stomach size (Peter & Valkenburg, 2014). Although these studies suggested that pornography use frequency might be associated with body image dissatisfaction, findings from the current study did not support these notions. However, this discrepancy may be a result of the aforementioned studies’ focus on the level of satisfaction from specific body parts, and the sole focus on the frequency of pornography use.

In the current study, we found no significant association between pornography frequency and social body comparison or negative body image. However, problematic pornography use was associated with negative body image via men’s greater tendency to social comparison. These findings are not surprising, as many studies have indicated that watching pornography in and of itself might not be predictive of negative outcomes (Bőthe et al., 2020, 2021c). In fact, it has been suggested that problematic pornography use are those aspects that are responsible for the association between pornography use and various negative aspects such as sexual problems (Bőthe et al., 2021c; de Alarcón et al., 2019), condom-less sex (Wright et al., 2022), sexting (Landripet, 2016), and sexual aggression (Krahé et al., 2022; Landripet, 2016) due to the impulsivity and control issues. It is also possible that several important correlates associated with problematic viewing, such as shame, guilt, and self-criticism (Sassover et al., 2023), neuroticism (Egan & Parmar, 2013), and psychopathology (Kor et al., 2014), might account for why problematic pornography use has a significant effect on men’s body image and social body comparison, whereas pornography use frequency does not. Moreover, in the context of pornography problematic use, individuals may undergo an escalation in the consumption of more extreme content (Lewczuk et al., 2022a, 2022b). For instance, those with pornography problematic use might gravitate toward increasingly extreme pornography, featuring exaggerated body ideals. This escalation in content may help elucidate the association between pornography problematic use and body image issues in men.

Examining the model across both groups (i.e., heterosexual and sexual minority men) revealed that in both heterosexual and sexual minority men pornography use frequency was not related significantly either to social body comparison or body image. However, the association between social body comparison and negative body image was stronger in the case of sexual minority men than heterosexual men. It is possible that this is because sexual minority men’s body image is shaped by internalized homophobia, and stigma (Bianchi et al., 2017; Kimmel & Mahalik, 2005) within the gay community (Doyle & Engeln, 2014; Wood, 2014).

### Study Limitations and Future Research

The current study possesses several limitations that warrant careful consideration. Firstly, data collection relied on a convenience sample, potentially limiting the generalizability of our findings to the broader population. Secondly, the study employed self-report measures, introducing the possibility of response bias, including under- or overreporting and social desirability bias. Notably, in the context of pornography research, participants may be hesitant to disclose accurate information about their consumption habits. However, studies suggest that individuals tend to be more candid about their sexual behaviors and pornography use when responding to online, anonymous surveys (Griffiths, 2012). Thirdly, the cross-sectional design of the study precludes the inference of causal relationships among the variables under investigation. Experimental research on the relationship between problematic pornography use, body image, and social comparison is crucial for establishing causal relationships and exploring the mechanisms involved. Despite limited existing studies, the inconclusive nature emphasizes the need for further experimental exploration. This approach allows for controlled exposure to specific pornography content, assessing short-term effects, and examining interventions to mitigate negative impacts, offering practical insights for prevention and intervention strategies.

Another significant limitation pertains to the measurement of body image dissatisfaction, as the instrument used did not include questions directly addressing distortions commonly portrayed in pornography, such as those related to penis size. While our findings indicate a positive association between higher levels of problematic pornography use and increased social comparison among men, the specific nature of these comparisons remains indeterminate. Questions persist regarding the focal point of these comparisons, whether involving a comparison to the ideal male body, specific body parts such as genitals, or assessments of sexual performance or partners. Subsequent research endeavors are warranted to delve into these nuanced dimensions further.

Moreover, it is crucial to acknowledge the absence of comprehensive measures related to stigma in our study, including variables such as stress, body shame, and the internalization of prejudice and discrimination. This limitation presents opportunities for future investigations to augment the comprehensiveness of research in this domain. Subsequent studies should explore the association between the study’s variables through the lens of stigma theory and integrate stigma-related measures. Examining stigma-related measures could potentially reveal additional mechanisms (e.g., shame, stress) contributing to the association of problematic pornography use with negative body image and social body comparison, especially among sexual minority men.

Finally, future studies could enhance our understanding of this topic by examining the current model while accounting for various characteristics of pornography use, such as the content consumed or the motives for viewing pornography (Bőthe, et al., 2021a, 2021b, 2021c). Expanding the scope of investigation in these areas would contribute valuable insights to the existing literature on the association between problematic pornography use, body image, and social comparison.

### Conclusion

This study provides support for the associations of problematic pornography use, and body image, via social body comparison with and without controlling for psychological distress. Some differences were found between heterosexual and sexual minority men, with sexual minority men reporting significantly higher levels of pornography use frequency, problematic pornography use, perceived realism in pornography, social body comparison, negative body image, and more psychological distress than did heterosexual men. Among heterosexual and sexual minority men, problematic pornography use was positively related to social body comparison, which, in turn, was positively related to negative body image. Body image and social body comparison should be addressed in therapy for problematic pornography use, and differences between heterosexual men and sexual minority men should be taken into account.

## References

[CR1] Baams, L., Overbeek, G., Dubas, J. S., Doornwaard, S. M., Rommes, E., & van Aken, M. A. G. (2015). Perceived realism moderates the relation between sexualized media consumption and permissive sexual attitudes in Dutch adolescents. *Archives of Sexual Behavior,**44*(3), 743–754. 10.1007/s10508-014-0443-7 25501659 10.1007/s10508-014-0443-7

[CR2] Barlett, C. P. (2008). Meta-analyses of the effects of media images on men’s body-image concerns. *Journal of Social and Clinical Psychology*, *27*(3), 279–310. https://doi-org.ezproxy.haifa.ac.il/10.1521/jscp.2008.27.3.279

[CR3] Barnes, M., Abhyankar, P., Dimova, E., & Best, C. (2020). Associations between body dissatisfaction and self-reported anxiety and depression in otherwise healthy men: A systematic review and meta-analysis. *PLoS ONE,**15*(2). 10.1371/journal.pone.0229268 10.1371/journal.pone.0229268 PMC7041842 32097427

[CR4] Beaulieu, V., Zippan, N., Nagy, L., Koós, M., Demetrovics, Z., Kraus, S. W., Potenza, M. N., International Sex Survey Consortium, & Bőthe, B. (2023). *Myths and facts about pornography [Infographic]*. Université de Montréal, Montréal, Canada.

[CR5] Bianchi, M., Piccoli, V., Zotti, D., Fasoli, F., & Carnaghi, A. (2017). The impact of homophobic labels on the internalized homophobia and body image of gay men: The moderation role of coming-out. *Journal of Language and Social Psychology,**36*(3), 356–367. 10.1177/0261927X16654735 10.1177/0261927X16654735

[CR6] Binnie, J., & Reavey, P. (2020). Problematic pornography use: Narrative review and a preliminary model. *Sexual and Relationship Therapy,**35*(2), 137–161. 10.1080/14681994.2019.1694142 10.1080/14681994.2019.1694142

[CR7] Bőthe, B., Nagy, L., Koós, M., Demetrovics, Z., Potenza, M. N., Demirgül, S. A., Gaudet, É., Ballester-Arnal, R., Batthyány, D., Bergeron, S., Billieux, J., Briken, P., Burkauskas, J., Cárdenas-López, G., Carvalho, J., Castro-Calvo, J., Chen, L., Ciocca, G., Corazza, O., ... Van Hout, M. C. (2024). Problematic pornography use across countries, genders, and sexual orientations: Insights from the International Sex Survey and comparison of different assessment tools. *Addiction*, *119*(5), 928–950. 10.1111/add.16431 38413365

[CR8] Bőthe, B., Tóth-Király, I., Bella, N., Potenza, M. N., Demetrovics, Z., & Orosz, G. (2021a). Why do people watch pornography? The motivational basis of pornography use. *Psychology of Addictive Behaviors,**35*(2), 172–186. 10.1037/adb0000603 32730047 10.1037/adb0000603

[CR9] Bőthe, B., Tóth-Király, I., Demetrovics, Z., & Orosz, G. (2021b). The short version of the problematic Pornography Consumption Scale (PPCS-6): A reliable and valid measure in general and treatment-seeking populations. *Journal of Sex Research,**58*(3), 342–352. 10.1080/00224499.2020.1716205 31995398 10.1080/00224499.2020.1716205

[CR10] Bőthe, B., Tóth-Király, I., Griffiths, M. D., Potenza, M. N., Orosz, G., & Demetrovics, Z. (2021c). Are sexual functioning problems associated with frequent pornography use and/or problematic pornography use? Results from a large community survey including males and females. *Addictive Behaviors*, *112*. 10.1016/j.addbeh.2020.106603 10.1016/j.addbeh.2020.106603 32810799

[CR11] Bőthe, B., Tóth-Király, I., Potenza, M. N., Orosz, G., & Demetrovics, Z. (2020). High-frequency pornography use may not always be problematic. *Journal of Sexual Medicine,**17*(4), 793–811. 10.1016/j.jsxm.2020.01.007 32033863 10.1016/j.jsxm.2020.01.007

[CR12] Bőthe, B., Tóth-Király, I., Zsila, Á., Griffiths, M. D., Demetrovics, Z., & Orosz, G. (2018). The development of the Problematic Pornography Consumption Scale (PPCS). *Journal of Sex Research,**55*(3), 395–406. 10.1080/00224499.2017.1291798 28276929 10.1080/00224499.2017.1291798

[CR13] Bridges, A. J., Sun, C. F., Ezzell, M. B., & Johnson, J. (2016). Sexual scripts and the sexual behavior of men and women who use pornography. *Sexualization, Media, & Society,**2*(4). 10.1177/2374623816668275 10.1177/2374623816668275

[CR14] Carroll, J. S., Padilla-walker, L. M., Nelson, L. J., Olson, C. D., Barry, C. M., & Madsen, S. D. (2008). Generation XXX: Pornography acceptance and use among emerging adults. *Journal of Adolescent Research,**23*(1), 6–30. 10.1177/0743558407306348

[CR15] Chen, F. F. (2007). Sensitivity of goodness of fit indexes to lack of measurement invariance. *Structural Equation Modeling,**14*(3), 464–504. 10.1080/10705510701301834 10.1080/10705510701301834

[CR16] Chen, L., Jiang, X., Wang, Q., Bőthe, B., Potenza, M. N., & Wu, H. (2022). The association between the quantity and severity of pornography use: A meta-analysis. *Journal of Sex Research,**59*, 304–319. 10.1080/00224499.2021.1988500 10.1080/00224499.2021.1988500 34723731

[CR17] Cheung, G. W., & Rensvold, R. B. (2002). Evaluating goodness-of-fit indexes for testing measurement invariance. *Structural Equation Modeling: A Multidisciplinary Journal,**9*(2), 233–255. 10.1207/S15328007SEM0902_5 10.1207/S15328007SEM0902_5

[CR18] Cranney, S. (2015). Internet pornography use and sexual body image in a Dutch sample. *International Journal of Sexual Health,**27*(3), 316–323. 10.1080/19317611.2014.999967 26918066 10.1080/19317611.2014.999967 PMC4764134

[CR19] Dawson, K., Nic Gabhainn, S., & MacNeela, P. (2020). Toward a model of porn literacy: Core concepts, rationales, and approaches. *Journal of Sex Research,**57*(1), 1–15. 10.1080/00224499.2018.1556238 30624090 10.1080/00224499.2018.1556238

[CR20] de Alarcón, R., de la Iglesia, J. I., Casado, N. M., & Montejo, A. L. (2019). Online porn addiction: What we know and what we don’t—a systematic review. *Journal of Clinical Medicine*, *8*(1). 10.3390/jcm8010091 10.3390/jcm8010091 PMC6352245 30650522

[CR21] Dittmar, H., Halliwell, E., & Stirling, E. (2009). Understanding the impact of thin media models on women’s body-focused affect: The roles of thin-ideal internalization and weight-related self-discrepancy activation in experimental exposure effects. *Journal of Social and Clinical Psychology,**28*(1), 43–72. 10.1521/jscp.2009.28.1.43 10.1521/jscp.2009.28.1.43

[CR22] Downing, M. J., Schrimshaw, E. W., Scheinmann, R., Antebi-Gruszka, N., & Hirshfield, S. (2017). Sexually explicit media use by sexual identity: A comparative analysis of gay, bisexual, and heterosexual men in the United States. *Archives of Sexual Behavior,**46*(6), 1763–1776. 10.1007/s10508-016-0837-9 27709363 10.1007/s10508-016-0837-9

[CR23] Doyle, D. M., & Engeln, R. (2014). Body size moderates the association between gay community identification and body image disturbance. *Psychology of Sexual Orientation and Gender Diversity,**1*(3), 279–284. 10.1037/sgd0000049 10.1037/sgd0000049

[CR24] Duggan, S. J., & McCreary, D. R. (2004). Body image, eating disorders, and the drive for muscularity in gay and heterosexual men: The influence of media images. *Journal of Homosexuality,**47*(3–4), 45–58. 10.1300/J082v47n03_03 15451703 10.1300/J082v47n03_03

[CR95] Egan, V., & Parmar, R. (2013). Dirty habits? Online pornography use, personality, obsessionality, and compulsivity. *Journal of Sex & Marital Therapy*, *39*(5), 394–409 23577795 10.1080/0092623X.2012.710182

[CR25] Fardouly, J., & Vartanian, L. R. (2016). Social media and body image concerns: Current research and future directions. *Current Opinion in Psychology,**9*, 1–5. 10.1016/j.copsyc.2015.09.005 10.1016/j.copsyc.2015.09.005

[CR27] Festinger, L. (1954). A theory of social comparison processes. *Human Relations*, *7*(2), 117–140.

[CR28] Frederick, D. A., & Essayli, J. H. (2016). Male body image: The roles of sexual orientation and body mass index across five national U.S. studies. *Psychology of Men and Masculinity*, *17*(4), 336–351. 10.1037/men0000031

[CR29] Girouard, A., Dion, J., Bőthe, B., O’Sullivan, L., & Bergeron, S. (2021). Bullying victimization and sexual wellbeing in sexually active heterosexual, cisgender and sexual/gender minority adolescents: The mediating role of emotion regulation. *Journal of Youth and Adolescence,**50*(11), 2136–2150. 10.1007/s10964-021-01471-7 34228262 10.1007/s10964-021-01471-7

[CR30] Gleason, N., & Sprankle, E. (2019). The effects of pornography on sexual minority men’s body image: An experimental study. *Psychology and Sexuality,**10*(4), 301–315. 10.1080/19419899.2019.1637924 10.1080/19419899.2019.1637924

[CR31] Goldsmith, K., Dunkley, C. R., Dang, S. S., & Gorzalka, B. B. (2017). Pornography consumption and its association with sexual concerns and expectations among young men and women. *Canadian Journal of Human Sexuality,**26*(2), 151–162. 10.3138/cjhs.262-a2 10.3138/cjhs.262-a2

[CR32] Grabe, S., Ward, L. M., & Hyde, J. S. (2008). The role of the media in body image concerns among women: A meta-analysis of experimental and correlational studies. *Psychological Bulletin,**134*(3), 460–476. 10.1037/0033-2909.134.3.460 18444705 10.1037/0033-2909.134.3.460

[CR33] Griffiths, M. D. (2012). The use of online methodologies in studying paraphilias—A review. *Journal of Behavioral Addictions,**1*(4), 143–150. 10.1556/JBA.1.2012.4.1 26165601 10.1556/JBA.1.2012.4.1

[CR34] Griffiths, S., Mitchison, D., Murray, S. B., & Mond, J. M. (2018). Pornography use in sexual minority males: Associations with body dissatisfaction, eating disorder symptoms, thoughts about using anabolic steroids and quality of life. *Australian & New Zealand Journal of Psychiatry*, *52*, 339–348. 10.1177/0004867417728807 10.1177/0004867417728807 28891676

[CR35] Grubbs, J. B., Hoagland, K. C., Lee, B. N., Grant, J. T., Davison, P., Reid, R. C., & Kraus, S. W. (2020). Sexual addiction 25 years on: A systematic and methodological review of empirical literature and an agenda for future research. *Clinical Psychology Review,**82*. 10.1016/j.cpr.2020.101925 10.1016/j.cpr.2020.101925 33038740

[CR36] Grubbs, J. B., Kraus, S. W., & Perry, S. L. (2019). Self-reported addiction to pornography in a nationally representative sample: The roles of use habits, religiousness, and moral incongruence. *Journal of Behavioral Addictions,**8*(1), 88–93. 10.1556/2006.7.2018.134 30632378 10.1556/2006.7.2018.134 PMC7044607

[CR37] Hald, G. M. (2006). Gender differences in pornography consumption among young heterosexual Danish adults. *Archives of Sexual Behavior,**35*, 577–585. 10.1007/s10508-006-9064-0 17039402 10.1007/s10508-006-9064-0

[CR38] Hanna, E., Monique Ward, L., Seabrook, R. C., Jerald, M., Reed, L., Giaccardi, S., & Lippman, J. R. (2017). Contributions of social comparison and self-objectification in mediating associations between Facebook use and emergent adults’ psychological well-being. *Cyberpsychology, Behavior, and Social Networking,**20*(3), 172–179. 10.1089/cyber.2016.0247 28263683 10.1089/cyber.2016.0247

[CR39] Hargreaves, D. A., & Tiggemann, M. (2009). Muscular ideal media images and men’s body image: Social comparison processing and individual vulnerability. *Psychology of Men and Masculinity,**10*(2), 109–119. 10.1037/a0014691 10.1037/a0014691

[CR40] Herbenick, D., Fu, T. C., Wright, P., Paul, B., Gradus, R., Bauer, J., & Jones, R. (2020). Diverse sexual behaviors and pornography use: Findings from a nationally representative probability survey of Americans aged 18 to 60 years. *Journal of Sexual Medicine,**17*(4), 623–633. 10.1016/j.jsxm.2020.01.013 32081698 10.1016/j.jsxm.2020.01.013

[CR41] Holland, G., & Tiggemann, M. (2016). A systematic review of the impact of the use of social networking sites on body image and disordered eating outcomes. *Body Image,**17*, 100–110. 10.1016/j.bodyim.2016.02.008 26995158 10.1016/j.bodyim.2016.02.008

[CR42] Jang, K., Park, N., & Song, H. (2016). Social comparison on Facebook: Its antecedents and psychological outcomes. *Computers in Human Behavior,**62*, 147–154. 10.1016/j.chb.2016.03.082 10.1016/j.chb.2016.03.082

[CR43] Kimmel, S. B., & Mahalik, J. R. (2005). Body image concerns of gay men: The roles of minority stress and conformity to masculine norms. *Journal of Consulting and Clinical Psychology,**73*(6), 1185–1190. 10.1037/0022-006X.73.6.1185 16392992 10.1037/0022-006X.73.6.1185

[CR44] Kohut, T., Balzarini, R. N., Fisher, W. A., Grubbs, J. B., Campbell, L., & Prause, N. (2020). Surveying pornography use: A shaky science resting on poor measurement foundations. *Journal of Sex Research,**57*(6), 722–742. 10.1080/00224499.2019.1695244 31821049 10.1080/00224499.2019.1695244

[CR45] Koletić, G. (2017). Longitudinal associations between the use of sexually explicit material and adolescents’ attitudes and behaviors: A narrative review of studies. *Journal of Adolescence,**57*, 119–133. 10.1016/j.adolescence.2017.04.006 28433892 10.1016/j.adolescence.2017.04.006

[CR46] Krahé, B., Tomaszewska, P., & Schuster, I. (2022). Links of perceived pornography realism with sexual aggression via sexual scripts, sexual behavior, and acceptance of sexual coercion: A study with german university students. *International Journal of Environmental Research and Public Health*, *19*(1). 10.3390/ijerph19010063 10.3390/ijerph19010063 PMC8751040 35010321

[CR47] Kraus, S. W., Krueger, R. B., Briken, P., First, M. B., Stein, D. J., Kaplan, M. S., Voon, V., Abdo, C. H. N., Grant, J. E., Atalla, E., & Reed, G. M. (2018). Compulsive sexual behaviour disorder in the ICD-11. *World Psychiatry,**17*(1), 108–109. 10.1002/wps.20464 29352554 10.1002/wps.20464 PMC5775124

[CR48] Kraus, S. W., Potenza, M. N., Martino, S., & Grant, J. E. (2015). Examining the psychometric properties of the Yale-Brown Obsessive-Compulsive Scale in a sample of compulsive pornography users. *Comprehensive Psychiatry,**59*, 117–122. 10.1016/j.comppsych.2015.02.007 25732412 10.1016/j.comppsych.2015.02.007

[CR49] Kroenke, K., Spitzer, R. L., Williams, J. B. W., & Löwe, B. (2009). An ultra-brief screening scale for anxiety and depression: The PHQ–4. *Psychosomatics,**50*(6), 613–621. 10.1016/s0033-3182(09)70864-3 19996233 10.1016/s0033-3182(09)70864-3

[CR50] Kvalem, I. L., Træen, B., Lewin, B., & Štulhofer, A. (2014). Self-perceived effects of internet pornography use, genital appearance satisfaction, and sexual self-esteem among young Scandinavian adults. *Cyberpsychology*, *8*(4). 10.5817/CP2014-4-4

[CR51] Lam, B. C., & Chan, D.K.-S. (2007). The use of cyberpornography by young men in Hong Kong: Some psychosocial correlates. *Archives of Sexual Behavior,**36*(4), 588–598. 10.1007/s10508-006-9124-5 10.1007/s10508-006-9124-5 17186123

[CR52] Landripet, I. (2016). *Perceived pornography realism as a mediator of the association between pornography use and problematic sexual behavior among male adolescents.* 13th Congress of the European Federation of Sexology 2016.

[CR53] Leickly, E., Nelson, K., & Simoni, J. (2017). Sexually explicit online media, body satisfaction, and partner expectations among men who have sex with men: A qualitative study. *Sexuality Research and Social Policy,**14*(3), 270–274. 10.1007/s13178-016-0248-7 28979572 10.1007/s13178-016-0248-7 PMC5624736

[CR54] Lewczuk, K., Wizła, M., Glica, A., Potenza, M. N., Lew-Starowicz, M., & Kraus, S. W. (2022a). Withdrawal and tolerance as related to compulsive sexual behavior disorder and problematic pornography use - Preregistered study based on a nationally representative sample in Poland. *Journal of Behavioral Addictions,**11*(4), 979–993. 10.1556/2006.2022.00076 36269607 10.1556/2006.2022.00076 PMC9881655

[CR55] Lewczuk, K., Wójcik, A., & Gola, M. (2022b). Increase in the prevalence of online pornography use: Objective data analysis from the period between 2004 and 2016 in Poland. *Archives of Sexual Behavior,**51*(2), 1157–1171. 10.1007/s10508-021-02090-w 34750777 10.1007/s10508-021-02090-w PMC8888374

[CR56] McKee, A., Albury, K., & Lumby, C. (2008). *The porn report*. Melbourne University.

[CR57] McKee, A., Byron, P., Litsou, K., & Ingham, R. (2020). An interdisciplinary definition of pornography: Results from a global Delphi panel [Technical Report]. *Archives of Sexual Behavior,**49*(3), 1085–1091. 10.1007/s10508-019-01554-4 31549362 10.1007/s10508-019-01554-4 PMC7058557

[CR58] Meyer, I. H. (2003). Prejudice, social stress, and mental health in lesbian, gay, and bisexual populations: Conceptual issues and research evidence. *Psychological Bulletin,**129*(5), 674–697. 10.1037/0033-2909.129.5.674 12956539 10.1037/0033-2909.129.5.674 PMC2072932

[CR59] Miller, D. J., Raggatt, P. T. F., & McBain, K. (2020). A literature review of studies into the prevalence and frequency of men’s pornography use. *American Journal of Sexuality Education,**15*(4), 502–529. 10.1080/15546128.2020.1831676 10.1080/15546128.2020.1831676

[CR60] Myers, T. A., & Crowther, J. H. (2009). Social comparison as a predictor of body dissatisfaction: A meta-analytic review. *Journal of Abnormal Psychology,**118*(4), 683–698. 10.1037/a0016763 19899839 10.1037/a0016763

[CR61] Newman, D. A. (2014). Missing data: Five practical guidelines. *Organizational Research Methods,**17*(4), 372–411. 10.1177/1094428114548590 10.1177/1094428114548590

[CR62] O’Brien, K. S., Caputi, P., Minto, R., Peoples, G., Hooper, C., Kell, S., & Sawley, E. (2009). Upward and downward physical appearance comparisons: Development of scales and examination of predictive qualities. *Body Image,**6*(3), 201–206. 10.1016/j.bodyim.2009.03.003 19447692 10.1016/j.bodyim.2009.03.003

[CR63] Pachankis, J. E., Clark, K. A., Burton, C. L., Hughto, J. M. W., Bränström, R., & Keene, D. E. (2020). Sex, status, competition, and exclusion: Intraminority stress from within the gay community and gay and bisexual men’s mental health. *Journal of Personality and Social Psychology,**119*(3), 713–740. 10.1037/pspp0000282 31928026 10.1037/pspp0000282 PMC7354883

[CR64] Paquette, M.-M., Dion, J., Bőthe, B., Girouard, A., & Bergeron, S. (2022). Heterosexual, cisgender and gender and sexually diverse adolescents’ sexting behaviors: The role of body appreciation. *Journal of Youth and Adolescence*, 278–290. 10.1007/s10964-021-01568-z 10.1007/s10964-021-01568-z 35098426

[CR65] Paslakis, G., Chiclana Actis, C., & Mestre-Bach, G. (2022). Associations between pornography exposure, body image and sexual body image: A systematic review. *Journal of Health Psychology*, *27*, 743–760. 10.1177/1359105320967085 33107365 10.1177/1359105320967085

[CR66] Peter, J., & Valkenburg, P. M. (2006). Adolescents’ exposure to sexually explicit online material and recreational attitudes toward sex. *Journal of Communication,**56*(4), 639–660. 10.1111/j.1460-2466.2006.00313.x 10.1111/j.1460-2466.2006.00313.x

[CR67] Peter, J., & Valkenburg, P. M. (2014). Does exposure to sexually explicit Internet material increase body dissatisfaction? A longitudinal study. *Computers in Human Behavior,**36*, 297–307. 10.1016/j.chb.2014.03.071 10.1016/j.chb.2014.03.071

[CR68] Preacher, K. J., & Hayes, A. F. (2008). Asymptotic and resampling strategies for assessing and comparing indirect effects in multiple mediator models. *Behavior Research Methods,**40*(3), 879–891. 10.3758/BRM.40.3.879 18697684 10.3758/BRM.40.3.879

[CR69] Rissel, C., Richters, J., de Visser, R. O., McKee, A., Yeung, A., & Caruana, T. (2017). A profile of pornography users in Australia: Findings from the Second Australian Study of Health and Relationships. *Journal of Sex Research,**54*(2), 227–240. 10.1080/00224499.2016.1191597 27419739 10.1080/00224499.2016.1191597

[CR70] Ryding, F. C., & Kuss, D. J. (2020). The use of social networking sites, body image dissatisfaction, and body dysmorphic disorder: A systematic review of psychological research. *Psychology of Popular Media,**9*(4), 412–435. 10.1037/ppm0000264 10.1037/ppm0000264

[CR71] Saiphoo, A. N., & Vahedi, Z. (2019). A meta-analytic review of the relationship between social media use and body image disturbance. *Computers in Human Behavior,**101*, 259–275. 10.1016/j.chb.2019.07.028 10.1016/j.chb.2019.07.028

[CR94] Sassover, E., Abrahamovitch, Z., Amsel, Y., Halle, D., Mishan, Y., Efrati, Y., & Weinstein, A. (2023). A study on the relationship between shame, guilt, self-criticism and compulsive sexual behaviour disorder. *Current Psychology*, *42*(10), 8347–8355 10.1007/s12144-021-02188-3

[CR72] Schellenberg, B. J. I., Bailis, D. S., & Mosewich, A. D. (2016). You have passion, but do you have self-compassion? Harmonious passion, obsessive passion, and responses to passion-related failure. *Personality and Individual Differences,**99*, 278–285. 10.1016/j.paid.2016.05.003 10.1016/j.paid.2016.05.003

[CR73] Sevic, S., Ciprić, A., Buško, V., & Štulhofer, A. (2020). The relationship between the use of social networking sites and sexually explicit material, the internalization of appearance ideals and body self-surveillance: Results from a longitudinal study of male adolescents. *Journal of Youth and Adolescence,**49*(2), 383–398. 10.1007/s10964-019-01172-2 31802316 10.1007/s10964-019-01172-2

[CR74] Sharp, G., & Oates, J. (2019). Sociocultural influences on men’s penis size perceptions and decisions to undergo penile augmentation: A qualitative study. *Aesthetic Surgery Journal,**39*(11), 1253–1259. 10.1093/asj/sjz154 31107944 10.1093/asj/sjz154

[CR75] Short, M. B., Black, L., Smith, A. H., Wetterneck, C. T., & Wells, D. E. (2012). A review of internet pornography use research: Methodology and content from the past 10 years. *Cyberpsychology, Behavior and Social Networking,**15*(1), 13–23. 10.1089/cyber.2010.0477 22032795 10.1089/cyber.2010.0477

[CR76] Sun, C. F., Wright, P., & Steffen, N. (2017). German heterosexual women’s pornography consumption and sexual behavior. *Sexualization, Media, & Society,**3*(1), 1–12. 10.1177/2374623817698113 10.1177/2374623817698113

[CR77] Taylor, K. (2022). “I’ve got to put one side aside if I want to enjoy it”: Pornography, perceived reality, and pornography viewers’ negotiated pleasures. *Sexuality and Culture*, *26*, 1215–1234. 10.1007/s12119-021-09939-1 10.1007/s12119-021-09939-1

[CR78] Tiggemann, M., & Anderberg, I. (2020a). Muscles and bare chests on Instagram: The effect of influencers’ fashion and fitspiration images on men’s body image. *Body Image,**35*, 237–244. 10.1016/j.bodyim.2020.10.001 33157398 10.1016/j.bodyim.2020.10.001

[CR79] Tiggemann, M., & Anderberg, I. (2020b). Social media is not real: The effect of ‘Instagram vs reality’ images on women’s social comparison and body image. *New Media and Society,**22*(12), 2183–2199. 10.1177/1461444819888720 10.1177/1461444819888720

[CR80] Tiggemann, M., Martins, Y., & Kirkbride, A. (2007). Oh to be lean and muscular: Body image ideals in gay and heterosexual men. *Psychology of Men and Masculinity,**8*(1), 15–24. 10.1037/1524-9220.8.1.15 10.1037/1524-9220.8.1.15

[CR81] Tiggemann, M., & McGill, B. (2004). The role of social comparison in the effect of magazine advertisements on women’s mood and body dissatisfaction. *Journal of Social and Clinical Psychology,**23*(1), 23–44. 10.1521/jscp.23.1.23.26991 10.1521/jscp.23.1.23.26991

[CR82] Tiggemann, M., & Polivy, J. (2010). Upward and downward: Social comparison processing of thin idealized media images. *Psychology of Women Quarterly,**34*(3), 356–364. 10.1111/j.1471-6402.2010.01581.x 10.1111/j.1471-6402.2010.01581.x

[CR83] Tylka, T. L. (2015). No harm in looking, right? Men’s pornography consumption, body image, and well-being. *Psychology of Men and Masculinity,**16*(1), 97–107. 10.1037/a0035774 10.1037/a0035774

[CR84] Tylka, T. L., Bergeron, D., & Schwartz, J. P. (2005). Development and psychometric evaluation of the Male Body Attitudes Scale (MBAS). *Body Image,**2*(2), 161–175. 10.1016/j.bodyim.2005.03.001 18089184 10.1016/j.bodyim.2005.03.001

[CR85] van den Berg, P., Paxton, S. J., Keery, H., Wall, M., Guo, J., & Neumark-Sztainer, D. (2007). Body dissatisfaction and body comparison with media images in males and females. *Body Image,**4*(3), 257–268. 10.1016/j.bodyim.2007.04.003 18089272 10.1016/j.bodyim.2007.04.003

[CR86] Vogels, E. A. (2019). Loving oneself: The associations among sexually explicit media, body image, and perceived realism. *Journal of Sex Research,**56*(6), 778–790. 10.1080/00224499.2018.1475546 29920127 10.1080/00224499.2018.1475546

[CR87] Want, S. C. (2009). Meta-analytic moderators of experimental exposure to media portrayals of women on female appearance satisfaction: Social comparisons as automatic processes. *Body Image,**6*(4), 257–269. 10.1016/j.bodyim.2009.07.008 19716779 10.1016/j.bodyim.2009.07.008

[CR88] Weinrich, J. D. (2014). On the design, development, and testing of sexual identity questions: A discussion and analysis of Kristen Miller and J. Michael Ryan’s work for the National Health Interview Survey. *Journal of Bisexuality*, *14*(3–4), 502–523. 10.1080/15299716.2014.952052

[CR89] Whitfield, T. H. F., Rendina, H. J., Grov, C., & Parsons, J. T. (2018). Viewing sexually explicit media and its association with mental health among gay and bisexual men across the U.S. *Archives of Sexual Behavior,**47*(4), 1163–1172. 10.1007/s10508-017-1045-y 28884272 10.1007/s10508-017-1045-y PMC5842099

[CR90] Wood, M. J. (2014). The gay male gaze: Body image disturbance and gender oppression among gay men. *Journal of Gay & Lesbian Social Services,**17*(2), 43–62. 10.4324/9781315864044-10 10.4324/9781315864044-10

[CR91] Wright, P. J. (2021). Overcontrol in pornography research: Let it go, let it go… [Letter to the Editor]. *Archives of Sexual Behavior,**50*(2), 387–392. 10.1007/s10508-020-01902-9 33398701 10.1007/s10508-020-01902-9

[CR92] Wright, P. J., Herbenick, D., & Paul, B. (2022). Casual condomless sex, range of pornography exposure, and perceived pornography realism. *Communication Research*, *49*, 547–566. 10.1177/00936502211003765

[CR93] Wright, P. J., & Štulhofer, A. (2019). Adolescent pornography use and the dynamics of perceived pornography realism: Does seeing more make it more realistic? *Computers in Human Behavior,**95*, 37–47. 10.1016/j.chb.2019.01.024 10.1016/j.chb.2019.01.024

